# Influencing factors of attitudes towards death and demands for death education among community-dwelling Chinese older adults: a cross-sectional study

**DOI:** 10.1186/s12889-022-13655-2

**Published:** 2022-06-23

**Authors:** Lei Lei, Hongyan Zhao, Lijuan Ran, Lihua Wang, Yu Luo

**Affiliations:** 1 School of Nursing, Army Medical University / Third Military Medical University, No. 30 Gaotanyan Street, Shapingba District, Chongqing, 400038 People’s Republic of China; 2 Xiaolongkan Community Health Service Center, No.4 Xiaolongkan Street, Shapingba District, Chongqing, 400030 People’s Republic of China; 3 Southwest University Hospital, No. 2 Tiansheng Road, Beibei District, Chongqing, 400715 People’s Republic of China; 4 The First Affiliated Hospital of Army Medical University, No. 29 Gaotanyan Street, Shapingba District, Chongqing, 400038 People’s Republic of China

**Keywords:** Attitude to Death, Older Adults, China, Community-dwelling, Cross-sectional Study

## Abstract

**Background:**

Considering older adults are getting closer to the end-of-life and face death more directly. Attitudes to death not only affect the physical and mental health of older adults, but also affect their acceptance of hospice care, even the quality of death. This study aims to explore the status, influencing factors of attitudes toward death and demands of death education among the community-dwelling older adults in southwestern China.

**Methods:**

A cross-sectional survey was adopted to investigate 683 community-dwelling older adults in Chongqing, China. Non-parametric test and multiple linear regression analysis was used to explore the influencing factors of different attitudes toward death of older adults in community.

**Results:**

The multiple linear regression models showed that different dimensions of death attitudes were affected by one or more factors including number of diseases, discussion about life and death, marital status, and average income per month. And community-dwelling older adults have high level demand for death education.

**Conclusions:**

Under the taboo culture of death in China, this study is one of the few studies on the attitudes toward death and the demands for death education of the community-dwelling older adults. This study contributes to enrich the global death studies and provide reference for the death education for older adults.

**Supplementary Information:**

The online version contains supplementary material available at 10.1186/s12889-022-13655-2.

## Background

The world’s population is rapidly ageing and the growth of aged population will continue for the coming decades especially in developing countries [1]. As early as 2000, China has entered an aging society [2]. In 2020, the number of people aged 60 years and older was over 264 million accounting for 18.70% of the total population [3]. Because of aging at biological level, the capacity to maintain physical and mental health of older adults is in decline [4]. Hence, most of the older adults have to face a growing risk of diseases, which ultimately lead to death. From the perspective of age, older adults are the group closest to death, and they are more likely to experience the inevitable loss of loved ones [5]. Although death is a fact that everyone will face, it has more significance for older people.

Significantly, attitudes towards death are closely related to individual physical and mental health and well-being. Death anxiety is a predictor for the severity of mental diseases in psychopathology [6]. The death of siblings may precipitate depression in older adults [7]. Attitudes towards death have significant behavioural and emotional implications, especially for older adults [8]. Preparations for death and quality of death are also affected by attitudes towards death [9]. If older adults are desperately afraid or avoiding of death, they cannot do their advanced care planning or talk about their preferences for dying and death. Negative attitudes towards death may lead to missed opportunities for hospice and palliative care and good death [10] Given the important relationship between attitudes towards death and health, attitudes towards death and quality of death, it is necessary to understand the attitudes towards death of older adults.

China has a fast-aging rate and a large elderly population. There are few studies on attitudes towards death among older adults. In Chinese traditional culture, talking about death is often considered as a cultural taboo, and most Chinese people thus choose to adopt the avoidance coping strategies to reduce the awareness of death. Talking about death with older adults is thought to have an ominous implication and may bring a bad luck [11], and this kind of death-sensitive culture limits the development of hospice and palliative care [12, 13]. Attitudes towards death influenced the older adults’ lifestyle and quality of life invisibly [14]. They may not be able to achieve personal maturity if they fail to consider their own death [15].

Many studies focused on the attitudes towards death of health professionals [16, 17], medical students [18, 19] and cancer patients [20], while relatively little attention had been paid to older adults [21]. Moreover, the results of those studies are diverse and inconsistent, which may be caused by the different experiences, religious beliefs, and cultural backgrounds of the research objects, indicating that the perception of death is highly personalized [22]. Attitudes towards death is multidimensional including positive and negative [21]. It is necessary to comprehensively understand the attitudes towards death of older adults and analyse the relevant influencing factors. This will provide reference for the construction of death education and psychological intervention for older adults to reduce excessive death anxiety and fear [22].

Death education is a series of educational activities to impart knowledge and skills related to death and dying [23]. The purpose of death education is to make individuals accept that death is an inevitable part of the life cycle gradually, normalize death and loss and promote better death preparedness [24]. Researches showed that death education could improve quality of life and well-being by reducing the negative attitudes associated with death [25, 26]. Older adults are more likely to experience suffering and despair of death because they are close to this final stage of life [27]. Therefore, it is more urgent to carry out death education according to the characteristics and influencing factors of the older adults’ attitudes towards death. It is beneficial for older adults to overcome the negative emotions of death and dying, improve the meaning of life, and contribute to active aging and healthy aging [28]. This study aims to enrich the global death attitude researches as one of the few studies on the attitudes towards death among the older adults.

## Methods

### Participants

From June 2021, a cross-sectional survey was carried out in Chongqing, one of the four municipalities in China. Before recruiting participants, we categorized the nine administrative districts of Chongqing main urban areas into three levels according to the gross domestic product (GDP) per capita. Then one administrative district was selected from each level by simple random sampling. And investigations were conducted in 1–3 communities in each of the selected administrative districts by convenience sampling. Next, we conducted a questionnaire survey among the community-dwelling older adults whose age is over than 60 years and who have lived in the community for more than one year and are able to communicate effectively with researchers. And all the study participants were recruited by advertisement and they were voluntary to participate in this study and gave an oral informed consent. The exclusion criterias were as follows:(1) no fixed place of residence; (2) under 60 years old; (3) impaired cognitive function (according to the medical records), unable to communicate effectively; (4) other reasons for not participating in the study (such as diseases exacerbation, end-stage diseases, etc.);

### Ethical Consideration

This study has been approved by the Ethics Committee of the University (No. 06–03, 2021). The investigation was conducted in full accordance with the Helsinki Declaration. Before the survey, researchers fully informed the older adults of the purpose, significance, and contents of this study. It was up to the older adults to decide whether to participate in the survey. The survey was initiated after the oral informed consent was obtained. During the investigation, if the respondents feel any discomfort, they can withdraw from the investigation at any time without any penalty.

### Instruments

#### Sociodemographic form

This form was designed by ourselves, which included queries regarding gender, age, ethnicity, religion, marital status, education, average income per month, profession types before retirement, profession types of adult children, self-care ability (self-assessed by older adults to describe fundamental skills required to independently care for oneself, such as eating, bathing, and shopping), number of diseases, discussion about life and death (discussions such as wills, funerals, resuscitate orders, place of death preferences, advance directives, etc.), experience of others death or dying.

#### Death attitude profile-revised (DAP-R)

For the attitudes toward death, Chinese version of Death Attitude Profile-Revised (DAP-R) was adopted, a culture adjusted version based on the profile developed by Wong, Reker and Gesser in 1994 [29] and its reliability and validity can be guaranteed [30]. The Cronbach’s α is 0.94 in this study, with 32 items in total. The scale has five dimensions including fear of death (FD, 7 items), death avoidance (DA, 5 items), neutral acceptance of death (NAD, 5 items), approach acceptance of death (AAD, 10 items), and escape acceptance of death (EAD, 5 items). The score ranges from 1 to 5 means “strongly disagree” to “strongly agree”. The higher score indicates the stronger emotions of the specific each dimension death attitude such as fear, avoidance or neutral acceptance, and so on. The score < 2.5 indicates a low degree of identification for this dimension, 2.5 ~ 3.5 indicates a medium degree of identification, and > 3.5 indicates a high degree of identification [31].

#### Demand form for death education contents

This form was self-designed including a total of 10 items about life, death, advance directives, hospice care etc. The Cronbach’s α of this form is 0.96 in this study. The demand form for death education contents is designed to investigate and collect the life and death-related contents that older adults want to know, including but not limited to the 10 items in this study. Other demands of death education contents of older adults can also be supplemented. The study participants evaluated the demand quantity of each item according to their own reality. There are five options for each item according to the degree of demand, that is: “much needed”, “needed” and “general needed”, “not needed”, “not needed at all.” “Much needed”, “needed” and “general needed” items were all considered to be required by the study participants, while the other options were not required.

### Data collection

The surveys were conducted pen-and-paper. Initially, community health service center staff and hospital staff helped to put up informational posters in different communities and made oral announcements to the community’s older adults to recruit enough participants. Then these local contacts kept in touch with potential participants and arranged a place and time for the researchers to meet. After the participants were selected through the inclusion and exclusion criteria, the first author explained the purpose, content, significance, and precautions of the survey to them. After providing oral informed consent, the participants were administered a structured paper-based questionnaire. During the survey, face-to-face questionnaire interviews were conducted by the uniformly trained and qualified research members. The interviewers read questionnaires to the participants item by item and recorded their answers. After confirming that there were no omissions, the interviewer signed and collected the questionnaire. A total of 718 questionnaires were distributed, and 683 valid questionnaires were collected, excluding those that were not answered completely. The response rate was 95.13%.

### Data analysis

Epidata 3.1 was used to check and input data, and SPSS 24.0 was used for data analysis. The measurement data were expressed as mean ± standard deviation, and the counting data were expressed as frequency and percentage. Non-parametric test was used for the comparison between groups for univariate analysis, and multiple linear regression analysis was adopted to explore the influencing factors. The different dimensions of death attitudes were taken as the dependent variables in both univariate analysis and multiple linear regression analysis, though the dependent variables were not normally distributed, the assumption of normality of the residuals was verified. (The verification results of the normality of the residuals are detailed in the supplementary-1). The data still met the requirements of multiple linear regression [32, 33]. Then the statistically significant factors in univariate analysis were taken as the independent variables in the multiple linear regression analysis. The parameter for entering or removing variables was set to “Stepwise” (Criteria: probability-of-F-to-enter <  = 0.05, probability-of-F-to-remove >  = 0.10). There is no collinearity between variables, and multiple linear regression analysis can be carried out. *P* < 0.05 was defined as statistically significant.

## Findings

A total of 683 older adults were surveyed in this study, among which 42.02% were male and 57.98% were female, with an average age of 73.48 ± 7.93 years (60–98 years). More than half of the older adults were married, but another 21.83% were widowed. The educational level of the participants in the study was mostly concentrated in primary, junior and senior/secondary school. Most of the respondents worked as farmers or workers before retirement. More than 77% of the older adults in this study suffered from chronic diseases and about 92% of older adults have experienced the death of someone else. But fewer than half of older adults have discussed life and death topics. For more details, see Table 1.

**Table 1:** Socio-demographic data of community-dwelling older adults (*n* = 683)

Groups	Number	Percent	Groups	Number	Percent
**Gender**			**Profession types before retirement**		
Male	287	42.02%	Agriculture	143	21.22%
Female	396	57.98%	Industry	322	47.77%
**Age (years)**			Government	26	3.86%
60–69	244	35.72%	Enterprise	76	11.28%
70–79	261	38.21%	Education	56	8.31%
Over 80	178	26.06%	Medicine	33	4.90%
**Ethnicity**			Others	18	2.67%
Han	678	99.27%	**Profession types of adult children**		
Other minorities	5	0.73%	Agriculture	15	2.26%
**Religion**			Industry	246	37.05%
Yes	31	4.71%	Government	35	5.27%
No	627	95.29%	Enterprise	138	20.78%
**Marital status**			Education	106	15.96%
Married	481	70.94%	Medicine	43	6.48%
Unmarried	5	0.74%	Others	81	12.20%
divorced	32	4.72%	**Self-care ability**		
Widowed	148	21.83%	Totally by self-care	573	85.78%
Remarried	12	1.77%	Need others help	82	12.28%
**Education**			Totally by others help	13	1.95%
Illiteracy	53	7.82%	**Number of deseases**		
Primary	213	31.42%	0	109	22.71%
Junior	186	27.43%	1	216	45.00%
Senior / Secondary	133	19.62%	2	96	20.00%
College	51	7.52%	≥ 3	59	12.29%
Graduate	42	6.19%	**Discussion about life and death**		
**Average income per month**			Yes	297	43.48%
Under 150 $	35	5.23%	No	386	56.52%
150–449 $	185	27.65%	**Experience of others death or dying**		
450–749 $	321	47.98%	Yes	625	91.51%
Over 750 $	128	19.13%	No	58	8.49%

### Community-dwelling older adults’ attitudes towards death

Table 2 showed the scores of each dimension of the participants’ attitudes towards death. The highest total score for each dimension of death attitudes is approaching acceptance of death, which is 28.19 ± 6.83, and the lowest total score is 14.28 ± 3.57 of escaping acceptance of death. The dimension, neutral acceptance of death, scored the highest (3.35 ± 0.73), followed by death avoidance (2.94 ± 0.71), and fear of death scored the lowest (2.74 ± 0.73) from item scores.

**Table 2:** Attitudes towards death among community-dwelling older adults (*n* = 683)

Attitudes to Death	Min	Max	Total Mean ± S.D	Item Mean ± S.D
Fear of Death (FD)	7.00	35.00	19.18 ± 5.12	2.74 ± 0.73
Death Avoidance (DA)	5.00	25.00	14.71 ± 3.55	2.94 ± 0.71
Neutral Acceptance of Death (NAD)	5.00	25.00	16.75 ± 3.66	3.35 ± 0.73
Approach Acceptance of Death (AAD)	10.00	50.00	28.19 ± 6.83	2.83 ± 0.68
Escaping Acceptance of Death (EAD)	5.00	25.00	14.28 ± 3.57	2.86 ± 0.71

### Univariate analysis on the attitudes towards death

The results of univariate analysis showed that fear of death was affected by marital status (*P* < 0.05), self-care ability (*P* < 0.05), the number of diseases (*P* < *0.05*) and the discussion about life and death (*P* < 0.05). Death avoidance was affected by the number of diseases *(P* < *0.01*). Neutral acceptance of death was influenced by the discussion about life and death (*P* < *0.001*) and experience of others death or dying (*P* < 0.05). Approach acceptance of death was influenced by age (*P* < *0.01*) and the discussion about life and death (*P* < *0.001*). Escape acceptance of death was influenced by average income per month (*P* < *0.01*), number of diseases (*P* < *0.01*), and the discussion about life and death (*P* < *0.001*). For details in Table 3.

**Table 3:** Single-factor analysis of attitudes toward death among community-dwelling older adults

Groups	FD	DA	NAD	AAD	EAD
**Gender**					
Male	18.97 ± 5.24	14.62 ± 3.68	16.51 ± 3.92	27.55 ± 6.92	14.17 ± 3.68
Female	19.33 ± 5.04	14.77 ± 3.46	16.93 ± 3.45	28.65 ± 6.74	14.35 ± 3.49
Z/X^2^	0.51	0.15	1.23	3.19	0.01
P	0.48	0.70	0.27	0.07	0.93
**Age (years)**					
60–69	19.08 ± 5.30	14.70 ± 3.59	17.22 ± 3.68	28.11 ± 6.92	14.08 ± 3.82
70–79	19.02 ± 4.96	14.82 ± 3.52	16.65 ± 3.71	27.82 ± 7.00	14.30 ± 3.38
Over 80	19.53 ± 5.12	14.56 ± 3.58	16.27 ± 3.49	28.83 ± 6.43	14.52 ± 3.48
Z/X^2^	0.29	0.22	0.95	12.72	2.70
P	0.87	0.90	0.62	< 0.01	0.26
**Ethnicity**					
Han	19.17 ± 5.13	14.71 ± 3.56	16.76 ± 3.66	28.18 ± 6.84	14.27 ± 3.57
Other minorities	20.20 ± 4.92	14.40 ± 3.44	16.20 ± 3.11	29.00 ± 6.56	14.80 ± 3.35
Z/X^2^	0.01	0.39	0.42	0.13	0.00
P	0.91	0.53	0.52	0.72	0.98
**Religion**					
Yes	19.87 ± 5.33	14.42 ± 3.93	17.39 ± 3.03	30.68 ± 6.82	14.32 ± 3.73
No	19.17 ± 5.08	14.79 ± 3.47	16.71 ± 3.67	28.06 ± 6.80	14.30 ± 3.53
Z/X^2^	0.27	0.41	0.997	2.67	0.03
P	0.61	0.52	0.318	0.10	0.87
**Marital Status**					
Married	19.00 ± 5.24	14.69 ± 3.66	16.97 ± 3.78	28.05 ± 7.09	14.15 ± 3.74
Unmarried	20.20 ± 4.49	15.20 ± 3.90	16.00 ± 3.94	26.60 ± 6.84	14.00 ± 3.81
Divorced	20.16 ± 4.00	15.50 ± 2.45	15.91 ± 2.58	29.09 ± 4.58	14.75 ± 2.66
Widowed	19.71 ± 4.99	14.64 ± 3.46	16.29 ± 3.37	28.53 ± 6.57	14.64 ± 3.13
Remarried	16.17 ± 3.90	13.58 ± 3.23	16.50 ± 4.21	27.08 ± 4.91	13.42 ± 3.63
Z/X^2^	10.24	1.88	9.43	1.98	2.15
P	< 0.05	0.76	0.05	0.74	0.71
**Education**					
Illiteracy	18.77 ± 4.42	14.00 ± 3.33	16.23 ± 3.02	28.40 ± 5.52	14.15 ± 3.28
Primary	19.91 ± 4.83	14.91 ± 3.15	16.60 ± 3.06	28.70 ± 6.38	14.48 ± 3.10
Junior	18.80 ± 5.39	14.84 ± 3.67	16.89 ± 3.59	27.96 ± 7.28	14.11 ± 3.77
Senior / Secondary	18.47 ± 5.20	14.59 ± 4.02	17.01 ± 4.58	28.01 ± 7.50	14.41 ± 4.14
College	18.59 ± 5.51	14.20 ± 3.88	16.53 ± 4.37	26.02 ± 6.80	13.51 ± 3.73
Graduate	19.98 ± 4.57	14.74 ± 2.99	16.83 ± 3.27	28.88 ± 5.26	14.26 ± 2.67
Z/X^2^	6.13	2.13	4.95	9.41	3.58
P	0.29	0.83	0.42	0.10	0.61
**Occupation types before retirement**					
Agriculture	19.48 ± 4.94	14.59 ± 3.28	16.66 ± 2.88	28.44 ± 6.13	14.31 ± 3.26
Industry	19.27 ± 5.18	14.76 ± 3.73	16.59 ± 3.93	28.25 ± 7.12	14.45 ± 3.71
Government	20.08 ± 5.66	15.38 ± 3.81	17.81 ± 3.07	28.73 ± 6.70	13.69 ± 3.50
Enterprise	17.58 ± 5.00	14.72 ± 3.39	17.03 ± 3.44	26.76 ± 7.27	13.54 ± 3.61
Education	19.98 ± 4.49	14.70 ± 2.83	16.55 ± 3.62	29.48 ± 5.45	14.18 ± 3.02
Medicine	17.94 ± 6.07	14.45 ± 4.47	17.55 ± 5.05	25.61 ± 8.09	14.52 ± 4.45
Others	19.83 ± 4.99	14.17 ± 3.42	17.56 ± 2.98	30.61 ± 6.36	14.72 ± 3.53
Z/X^2^	10.43	2.34	10.15	12.11	7.35
P	0.11	0.89	0.12	0.06	0.29
**Occupation types of adult children**					
Agriculture	21.73 ± 4.18	15.07 ± 3.06	16.40 ± 3.09	30.93 ± 5.91	15.80 ± 2.73
Industry	19.57 ± 5.20	14.60 ± 3.76	16.60 ± 3.86	28.61 ± 7.04	14.56 ± 3.76
Government	19.80 ± 4.68	14.46 ± 3.32	17.43 ± 3.43	28.26 ± 5.95	13.34 ± 2.95
Enterprise	18.47 ± 4.93	14.84 ± 3.49	17.22 ± 3.62	27.87 ± 6.46	14.27 ± 3.70
Education	19.23 ± 5.37	15.42 ± 3.38	16.91 ± 3.06	28.03 ± 7.38	14.11 ± 3.45
Medicine	18.70 ± 6.12	13.60 ± 3.93	17.02 ± 4.52	27.49 ± 7.51	13.72 ± 4.00
Others	18.51 ± 4.89	14.53 ± 3.42	16.12 ± 3.59	27.16 ± 6.65	14.05 ± 3.24
Z/X^2^	8.53	8.50	8.06	5.21	8.96
P	0.20	0.20	0.23	0.52	0.18
**Average income per month**					
Under 150 $	19.94 ± 4.94	14.17 ± 3.54	16.11 ± 2.83	29.77 ± 4.77	15.06 ± 2.62
150–449 $	19.36 ± 5.08	15.02 ± 3.23	16.71 ± 3.57	28.39 ± 6.77	14.50 ± 3.70
450–749 $	19.19 ± 5.15	14.77 ± 3.74	16.77 ± 3.63	28.17 ± 6.90	14.43 ± 3.52
Over 750 $	18.56 ± 5.18	14.20 ± 3.49	16.91 ± 4.04	27.51 ± 7.17	13.36 ± 3.53
Z/X^2^	3.23	4.84	3.81	3.42	13.91
P	0.36	0.18	0.28	0.33	< 0.01
**Self-care ability**					
Totally by self-care	18.91 ± 5.25	14.62 ± 3.65	16.75 ± 3.77	28.05 ± 7.04	14.19 ± 3.63
Need others help	20.43 ± 4.52	15.20 ± 3.16	17.26 ± 3.12	28.44 ± 6.01	14.51 ± 3.42
Totally by others help	20.92 ± 2.22	15.31 ± 2.32	15.69 ± 2.50	31.08 ± 4.11	15.46 ± 3.07
Z/X^2^	9.05	2.22	2.94	2.16	1.03
P	< 0.05	0.33	0.23	0.34	0.60
**Number of deseases**					
0	17.75 ± 5.50	13.76 ± 3.98	16.59 ± 4.09	26.97 ± 7.40	13.29 ± 3.74
1	19.65 ± 5.01	14.99 ± 3.40	17.34 ± 3.19	28.62 ± 6.47	14.44 ± 3.34
2	19.65 ± 5.10	15.85 ± 3.23	17.55 ± 2.98	28.99 ± 6.48	14.96 ± 3.42
≥ 3	19.42 ± 6.15	15.12 ± 3.61	17.81 ± 3.67	28.66 ± 7.15	15.36 ± 3.76
Z/X^2^	7.96	15.85	2.71	4.225	14.55
P	< 0.05	< 0.01	0.44	0.24	< 0.01
**Discussion about life and death**					
Yes	19.75 ± 4.58	14.95 ± 3.21	17.40 ± 3.41	29.32 ± 6.34	14.98 ± 3.40
No	18.73 ± 5.47	14.52 ± 3.79	16.25 ± 3.77	27.31 ± 7.07	13.74 ± 3.61
Z/X^2^	5.04	0.69	12.81	13.31	17.61
P	< 0.05	0.41	< 0.001	< 0.001	< 0.001
**Experience of others death or dying**					
Yes	19.26 ± 5.09	14.74 ± 3.50	16.85 ± 3.62	28.35 ± 6.76	14.36 ± 3.53
No	18.22 ± 5.38	14.36 ± 4.09	15.72 ± 3.93	26.47 ± 7.46	13.43 ± 3.90
Z/X^2^	1.58	0.07	4.45	2.86	2.28
P	0.21	0.80	< 0.05	0.09	0.13

*Note*: Bonferroni correction has been done. *FD* Fear of Death, *DA* Death Avoidance, *NAD* Neutral Acceptance of Death, *AAD *Approach Acceptance of Death, *EAD* Escape Acceptance of Death

### Multiple linear regression analysis of attitudes toward death

According to the analysis, the multiple linear regression equations of community resident older adults’ death attitudes are finally established as follows. Y(FD) = 20.08–1.55*(Not sick)-3.97*(Remarried)-0.98*(No discussion about life and death)_._ According to the standardized regression coefficient analysis, the influence of “Not sick”, “Remarried”, “No discussion about life and death” on fear of death decreases successively. Y(DA) = 15.02–1.25*(Not sick) + 0.84*(Two diseases). According to the standardized regression coefficient analysis, “Not sick”has a greater impact on death avoidance than “Two diseases”. Y(NAD) = 17.40–1.15*(No discussion about life and death). Y(AAD) = 27.31 + 2.01*(Discussion about life and death). Y(EAD) = 14.37–1.24*(Not sick) + 1.10*(Discussion about life and death)-1.02*(Average income per month over 750 $). According to the comparison of standardized regression coefficients, “Discussion about life and death” has the greatest influence on escape acceptance death, followed by “Not sick”, and “Average income per month over 750 $” has the least influence on escape acceptance. For details in Tables 4 & 5.

**Table 4:** Multiple linear regression independent variable assignment of attitudes to death among community-dwelling older adults

**Variables**	**Assignment** (dummy variables)
X_1_: Age	60 ~ 69 years = 1, 70 ~ 79 years = 2, Over 80 years = 3
X_2_: Marital status	Married = 1, Unmarried = 2, Divorced = 3, Widowed = 4, Remarried = 5
X_3_: Average income per month	Under 150 $ = 1, 150–449 $ = 2, 450–749 $ = 3, Over 750 $ = 4
X_4_: Self-care ability	Totally by self-care = 1, Need others help = 2, Totally by others help = 3
X_5_: Number of deseases	Not sick = 0, One disease = 1, Two diseases = 2, Having three or more diseases = 3
X_6_: Discussion about life and death	Yes = 1, No = 2
X_7_: Experience of others death or dying	Yes = 1, No = 2

**Table 5:** Results of multiple linear regression of attitudes toward death among community-dwelling older adults

Death attitudes	Variables	B	SD	β	t	p	Tolerance	VIF	F	P
FD	Constant	20.08	0.36		55.74	< 0.001			6.10	< 0.001
	Not sick	-1.55	0.59	-0.12	-2.64	< 0.05	0.96	1.04		
	Remarried	-3.97	1.76	-0.10	-2.25	< 0.05	1.00	1.00		
	No discussion about life and death	-0.98	0.49	-0.09	-2.00	< 0.05	0.96	1.04		
DA	Constant	15.02	0.21		70.55	< 0.001			9.33	< 0.001
	Not sick	-1.25	0.40	-0.15	-3.14	< 0.01	0.93	1.08		
	Two diseases	0.84	0.42	0.09	2.01	< 0.05	0.93	1.08		
NAD	Constant	17.40	0.21		66.30	< 0.001			17.06	< 0.001
	No discussion about life and death	-1.15	0.28	-0.16	-4.13	< 0.001	1.00	1.00		
AAD	Constant	27.31	0.34		79.34	< 0.001			14.82	< 0.001
	Discussion about life and death	2.01	0.52	-0.15	3.85	< 0.001	1.00	1.00		
EAD	Constant	14.37	0.26		55.41	< 0.001			10.39	< 0.001
	Not sick	-1.24	0.39	-0.15	-3.18	< 0.01	0.96	1.04		
	Discussion about life and death	1.10	0.33	0.15	3.35	< 0.01	0.96	1.05		
	Average income per month over 750 $	-1.02	0.41	-0.11	-2.52	< 0.05	0.99	1.01		

*Note*: *B* unstandardized coefficient, *SD* standard deviation, *β* standardized coefficient, *t* test statistics of each variable, *p* test significance level of each variable, *VIF* Variance Inflation Factor, *F* test statistics of each regression equation, *P* test significance level of each regression equation, *FD* Fear of Death, *DA* Death Avoidance, *NAD* Neutral Acceptance of Death, *AAD* Approach Acceptance of Death, *EAD* Escape Acceptance of Death

### Demands for the contents of death education

**Fig. 1:**
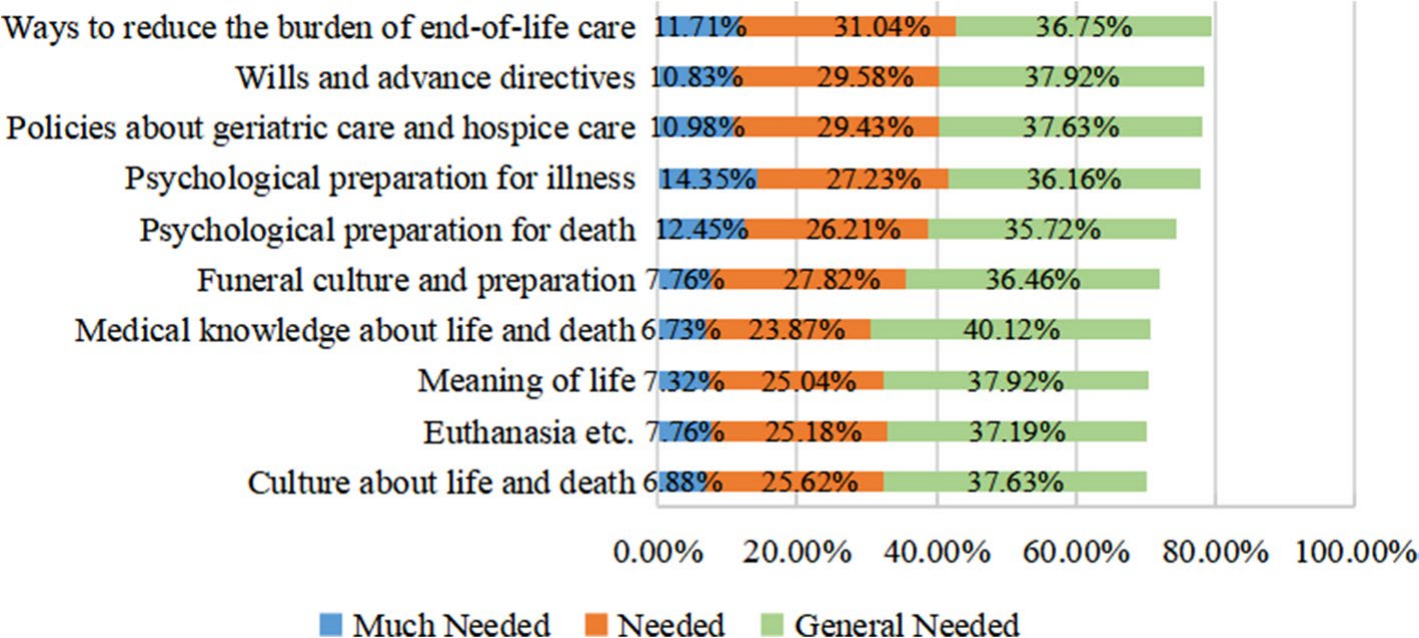
Demands for contents on death education of community-dwelling older adults

More than 70% of the community-dwelling older adults in this study had demands for death education. The highest percent items of overall needed were “ways to reduce the burden of end-of-life care, wills and advance directives, policies about geriatric care and hospice care, psychological preparation for illness” which the proportions were close to 80%. “Medical knowledge about life and death, culture about life and death, euthanasia etc., and meaning of life” are the minimum required items. For details in Fig. 1.

## Discussion

With an increase in aging population, the health of older adults has become one of the main public health issues in China [34]. Considering older adults are getting close to the end of their lives and more susceptible to diseases, as well as experiencing the loss of others inevitably [5], it is necessary to know their attitudes towards death and demands for death education. This study aims to take death as a window to a better life and help older adults to think about their life plans. It is helpful for maximizing the social value of older adults and realizing healthy and active aging. In the context of a traditional Chinese culture in which talking about death is a taboo [13], this study is one of the few studies on the attitudes towards death and their demands for death education in the community dwelling older adults. The findings may provide support for community health education and hospice and palliative care promotion.

### Attitudes towards death among the community-dwelling older adults

A study of death anxiety among older adults in the UK shows that anxiety levels decrease with age [35]. The Korean older adults have a slightly positive attitude towards death, which is consistent with the results of this study [36]. In this study, the score of neutral acceptance of death among the older adults was the highest, but still lower than positive attitude towards death (3.45 ± 0.58) among the older adults in Taiwan, China [37]. The results of this study showed that the attitude towards death in the study participants was neutral acceptance of death mainly. Although the education level of the older adults in this survey is not high, 66.67% had not receive education higher than senior school, and all the participants in this survey had not receive death education before, they can still view death as a stage of life rationally. This may be related to the participants’ life experience and psychosocial development.

The score of death avoidance ranked second. To some extent, it shows that although the study participants can accept death as a part of life, most of them still avoid talking about death-related issues, such as planning before dying. Avoiding talking about death is a way to reduce the awareness of death, but it also reduces the acceptance of hospice and palliative care [38], and the quality of death [39]. In addition, avoiding talking about death may put the older adults who have experienced the death of their loved ones in a long period of sadness, even depression or other psychological diseases [40], which is not conducive to their mental health [41]. This is something that health professionals need to pay attention to.

The scores of escape acceptance and approach acceptance toward death in the older adults were close, and both were higher than the theoretical median 2.5, indicating that when older adults have to face death, they can accept death from the point of view of reality and/or their longing, rather than extreme fear.

Fear of death scored lowest in this study is different from that in Iran. Death anxiety among older Iranians was relatively high [42]. Nearly 80% of the community-dwelling older adults in this study suffer from chronic diseases but 98.05% have no care problems according to their self-assessments. It may be that the older adults have less near-death feelings or experiences, and they also avoid the topic of death, so their fear and anxiety are relatively low.

The study by Lockhart, L. K. et al. showed that the score of approach acceptance of death in American older adults was 3.94 ± 0.93, which was higher than the 2.83 ± 0.68 in this study, while the scores of escaping acceptance of death and fear of death were 2.52 ± 0.68 and 2.15 ± 0.58 respectively, which were lower than those in this study [43]. It showed that the characteristics of death attitudes of the older adults in the United States are more clear than that of the older adults in this study. It is worth noting that the scores of attitudes towards death in all dimensions are between 2.5 and 3.5, indicating that the attitudes towards death of community-dwelling older adults are complex and multi-dimensional. No obvious attitude tendency towards death in older adults indicates that the cognition towards death is still in a vague stage, and professional guidance and education are required.

### Influencing factors of attitudes towards death among the community-dwelling older adults

Many studies have come to different findings about the influencing factors of attitudes towards death [15, 44], and this is related to the unique cultural background of the research objects [45]. In Chinese culture, the study community-dwelling older adults’ attitudes towards death were influenced by the number of diseases, discussion about life and death, marital status, and average income per month.

The item “number of diseases” has an impact on fear of death, death avoidance and escape acceptance of death. When the investigated group was not sick, the scores of fear of death, death avoidance and escape acceptance of death would decrease. This indicates that diseases will bring changes in the older adults’ thoughts and attitudes towards death. When the number of diseases increased, the negative effect on fear of death, death avoidance and escape acceptance of death disappeared, indicating that the presence or absence of diseases is the key to affect attitudes towards death in older adults. Only when the number of diseases increased to two, there was a positive impact on death avoidance. With the number of diseases increasing, older adults may be aware of the proximity of death, deepening their avoidance of death. However, when the number of diseases increased beyond two, the positive and negative effects on attitudes towards death all disappeared. The above results suggest that community health professionals should pay more attention to the emotional and psychological changes of the older adults when the diseases are first diagnosed and the number of diseases increases to two.

The discussion about life and death is the factor that affects fear of death, neutral acceptance of death, approaching acceptance of death and escape acceptance of death. The discussion about life and death has a positive effect on the community-resident older adults’ scores of approaching acceptance of death and escape acceptance of death. The life and death relevant discussions increase older adults’ recognition of approaching acceptance of death and escape acceptance of death. The scores of fear of death and neutral acceptance of death will decrease if there is no discussion about life and death. Overall, the life and death discussion has an impact on all dimensions of the community-dwelling older adults’ attitudes towards death except death avoidance. The discussion about life and death is a double-edged sword, which should be carried out cautiously. On one hand, it may generally improve the awareness of death and promote older adults to accept death neutrally to some extent. On the other hand, it may also arise fear of death, approach acceptance of death and escape acceptance of death. Therefore, more in-depth, and detailed research is needed in how to standardize or appropriately carry out the life and death discussion within older adults.

The effect of remarriage on fear of death of community-dwelling older adults is negative. Marital status was also an influencing factor in cancer patients’ death anxiety [46]. The proportion of remarried older adults is relatively low, but marital status is statistically significant in both univariate and multivariate regression analysis of fear of death. Older adults who were remarried had a lower score of fear of death and were less afraid of death than older adults in other marital status. This may be due to an unhappy life experience and the courage to make a change of remarried older adults. To some extent, remarriage shows older people’s ability to integrate into life and find happiness again [47], and this experience may reduce the feelings of fear when they are in face of death.

This study found that an average monthly income of more than 750 $ had a negative effect on the score of escape acceptance of death for the study participants. Escape acceptance of death refers to that death is accepted as a way of escaping from a life of misery [9]. High income can improve the quality of life. An average monthly income of more than 750 $ can provide a better guarantee for the real life needs of older adults in China. Hence, it is possible that the older people at this income level do not find life painful and the tendency to escape acceptance of death is weak.

In addition to the above factors that had statistical significance in the multiple linear regression analysis, age, self-care ability and experience of others death or dying were statistically significant in the univariate analysis of death attitudes of the community-dwelling older adults in this study.

Age was a significant influencing factor on the attitudes towards death between the young and old groups in many studies [8, 14]. In this study, the attitudes towards death among the young-old (60–69 years old), the middle-old (70–79 years old) and the old-old (over 80 years old) population differ only in the approach acceptance of death dimension. The score of approach acceptance of death among the middle-old is the lowest, which may be because the physical function of the middle-old is weaker than that of the young-old, and the psychological preparation for death is not sufficient when compared with that of the old-old. However, in the multivariate analysis, age did not enter any regression equation of death attitudes. So, under the comprehensive influence of multiple social factors, age of older adults has no significant impact on their attitudes towards death. Older adults in this study with different self-care abilities have different degrees of fear of death. Those who are fully capable of self-care have the lowest score of fear of death, but it is not statistically significant in multivariate analysis. Older people with the slow reduction of self-care ability may have a better death preparation compared with receiving a sudden shocking diagnosis, so it is not a significant factor in a comprehensive setting. Chronic, or severe illnesses seriously affect older adults’ acceptance of death [22]. The experience of others death or dying was also an influencing factor in the univariate analysis of death attitudes of the community-dwelling older adults. By witnessing other people’s death, the community-dwelling older adults may have a higher score of neutral acceptance of death, indicating that the inevitable experience of witnessing the death of others unconsciously acts as a reminder of death and makes older adults realize that death is a natural law [48].

### Demands for the content of death education within the community-dwelling older adults

Through analysing the influencing factors of death attitudes, we found that the discussion of life and death is an important way for community health professionals to improve the awareness about death and dying and affect death attitudes of older adults. In fact, if we want to make the best of the positive guidance of such discussion, professional and scientific death education need to be carried out for older adults. Death education is the opportunity and window for discussing life and death for Chinese older adults [28]. Therefore, this study surveyed the contents of death education that the community-dwelling older adults are interested in and demanded.

In terms of the overall degree of demands, the percent for ways to reduce the burden of end-of-life care is the highest. The demands proportion of “wills and advance directives, policies about geriatric care and hospice care, psychological preparation for death” followed, indicating that older adults in this study hope to plan their terminal stage, improve the quality of death effectively, and achieve a good death. The community-dwelling older adults in this study have relatively low demands for medical knowledge, meaning of life, euthanasia (not allowed by Chinese law [49]) and culture about life and death. The degree of demands for the ten death education items in this study were all more than 70%, and this means that the study participants are eager to know more about death and hope to communicate with others about life and death [12].

However, there is no universal death education for the community-dwelling older adults in China. Although there have been a few death education studies for cancer patients [50], health professionals [51] and medical students [52], the community-dwelling older adults have been neglected. Different from the developed countries such as United States, United Kingdom and Canada, where death education has become universal in primary and secondary schools [24, 53], death education in China is carried out only in older adults sporadically [54]. In view of sociocultural traditions, mention of death is still considered sensitive or emotionally damaging, especially when discussing death with older adults. Therefore, the content design of death education for community-dwelling older adults should pay more attention to the actual demands of them. In this way, death anxiety of older adults can be avoided as far as possible, and at the same time, older adults can be motivated to think about death, plan their end-of-life care, and cherish the present time, thus improving the quality of life and acquiring happiness [55].

### Limitations

The proportion of some groups varies greatly such as ethnicity, religions, and marital status. But this is also the reality in China. Influenced by the research contents, older adults who are very sensitive to the topic of death or extremely afraid of death may be reluctant to participate in this study. In fact, this is difficult to avoid under the premise that the survey respondents voluntarily participate in the death attitude survey. And this study was completed in a small sample in Chongqing, southwest China, replicating it in an area where random sampling could be used is recommended. Future studies should expand the sample size and maximize the characteristics of different older adults’ groups, to better explore the factors affecting attitudes towards death.

## Conclusion

This study is one of the few studies on the attitude towards death and the demands for death education of Chinese community-dwelling older adults. The attitudes towards death of older adults are complex and multi-dimensional, and the number of diseases, discussion about life and death, marital status, and average income per month were the influencing factors. The community-dwelling older adults are interested in the contents of death education and demand them, such as psychological preparations for illness, ways to reduce the burden of end-of-life care, wills and living wills. The findings of this study contribute to enrich the global death attitude studies and provide reference for the development of death education for older adults, which are helpful to reduce the psychological burden and improve the quality of death for older adults.

## Supplementary Information

**Additional file 1. **

## Data Availability

The data that support the findings of this study are available from the corresponding author upon reasonable request.
