# Supplementary material for: Influencing factors of attitudes towards death and demands for death education among community-dwelling Chinese older adults: a cross-sectional study
Source: BMC Public Health. 2022 Jun 23;22:1242. doi: 10.1186/s12889-022-13655-2 (PMC9219144; doi:10.1186/s12889-022-13655-2)
Supplement: Supplementary file 1 [file 12889_2022_13655_MOESM1_ESM.doc]

Histogram

Dependent Variable: FD

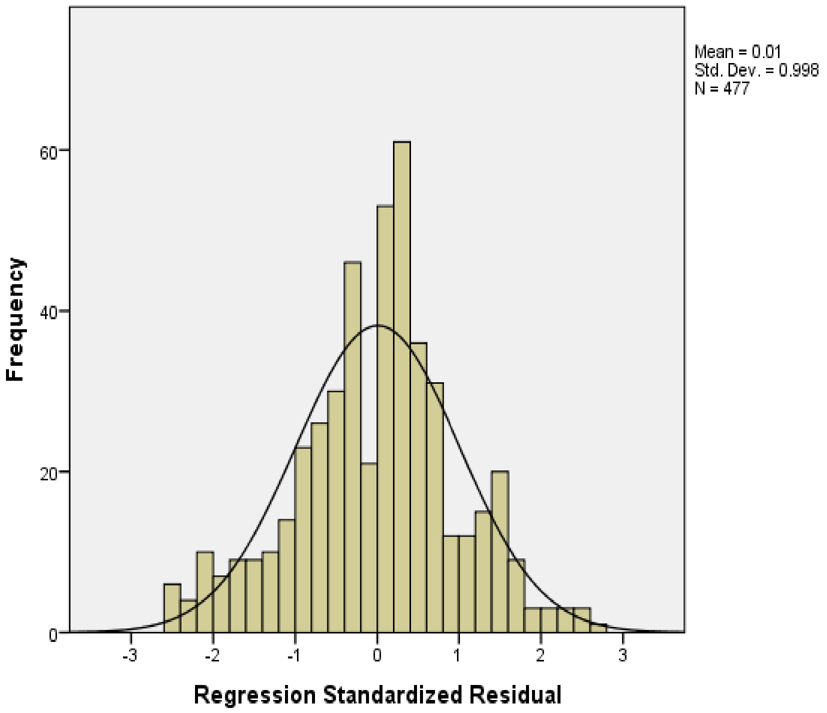

Normal P-P Plot of Regression Standardized Residual

Dependent Variable: FD

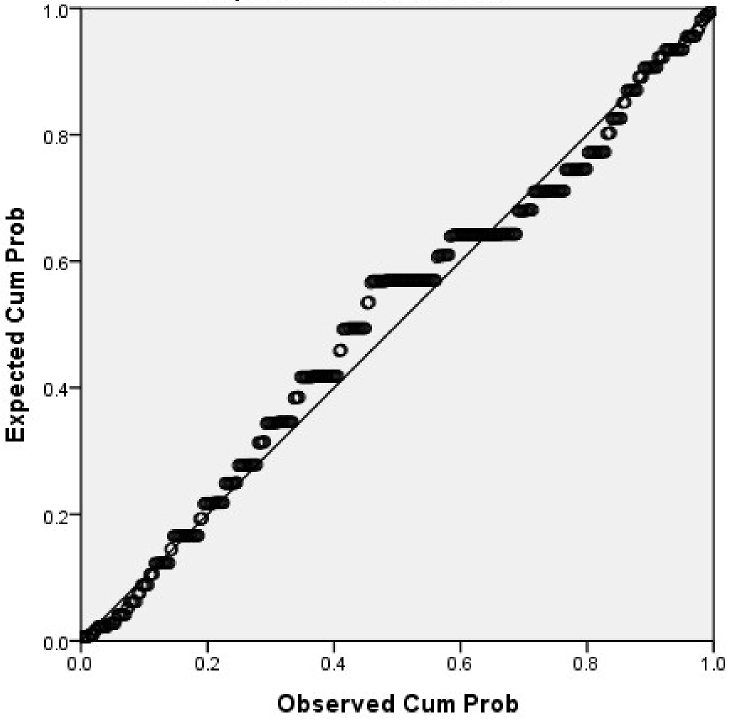

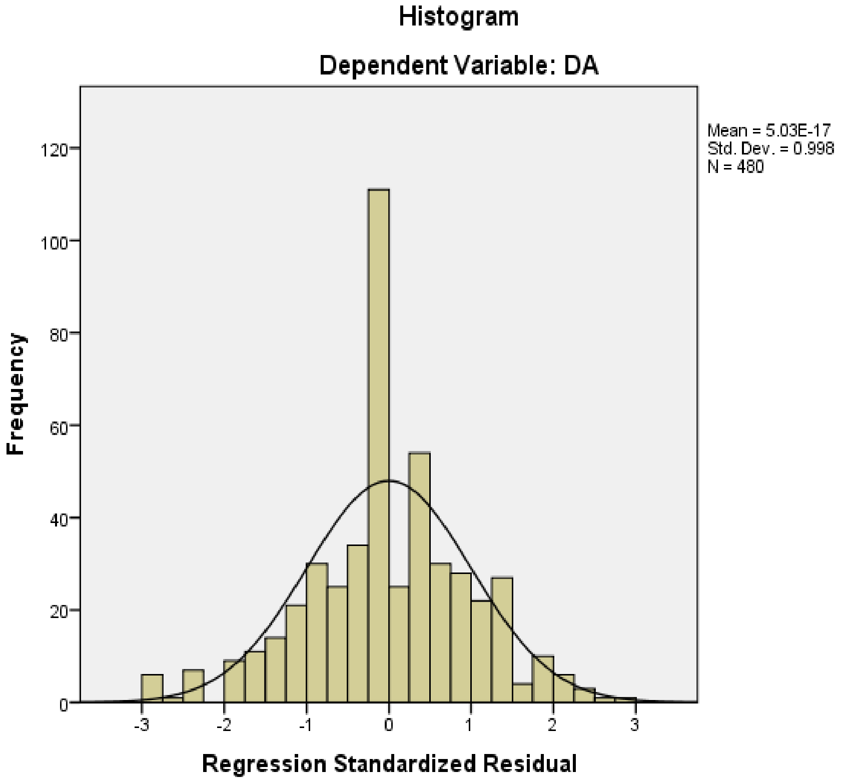
Histogram
Dependent Variable: DA
Mean = 5.03E-17
Std. Dev. = 0.998
N = 480
Frequency
120
100
80
60
40
20
0
-3
-2
-1
0
1
2
3
Regression Standardized Residual

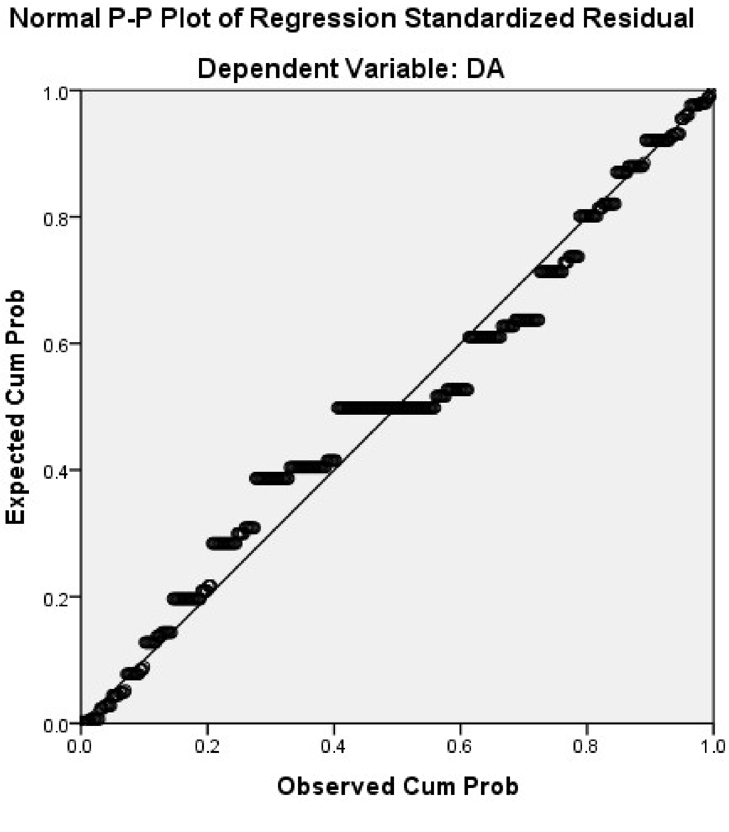
Normal P-P Plot of Regression Standardized Residual
Dependent Variable: DA
Expected Cum Prob
1.0
0.8
0.6
0.4
0.2
0.0
0.0
0.2
0.4
0.6
0.8
1.0
Observed Cum Prob

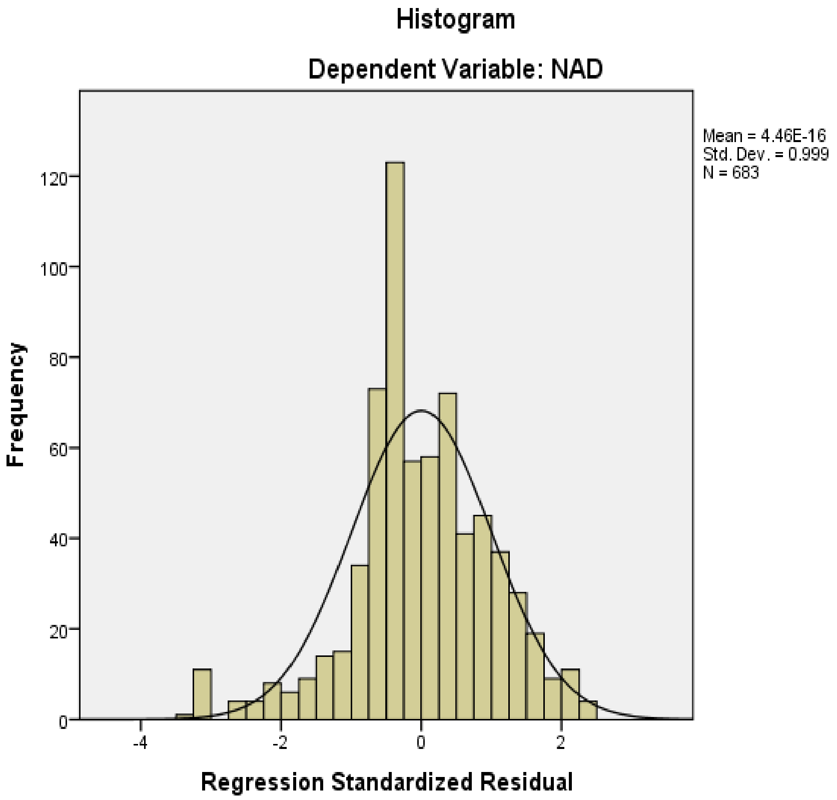
Histogram
Dependent Variable: NAD
Mean = 4.46E-16
Std. Dev. = 0.999
N = 683
Frequency
0
20
40
60
80
100
120
-4
-2
0
2
Regression Standardized Residual

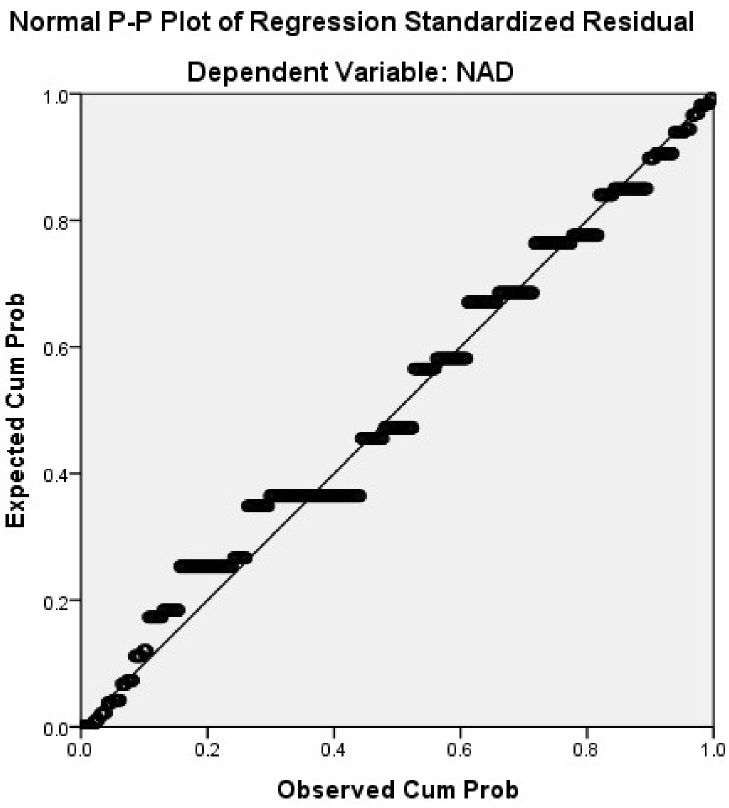
Normal P-P Plot of Regression Standardized Residual
Dependent Variable: NAD
Expected Cum Prob
0.0
0.2
0.4
0.6
0.8
1.0
0.0
0.2
0.4
0.6
0.8
1.0
Observed Cum Prob

Histogram

Dependent Variable: AAD

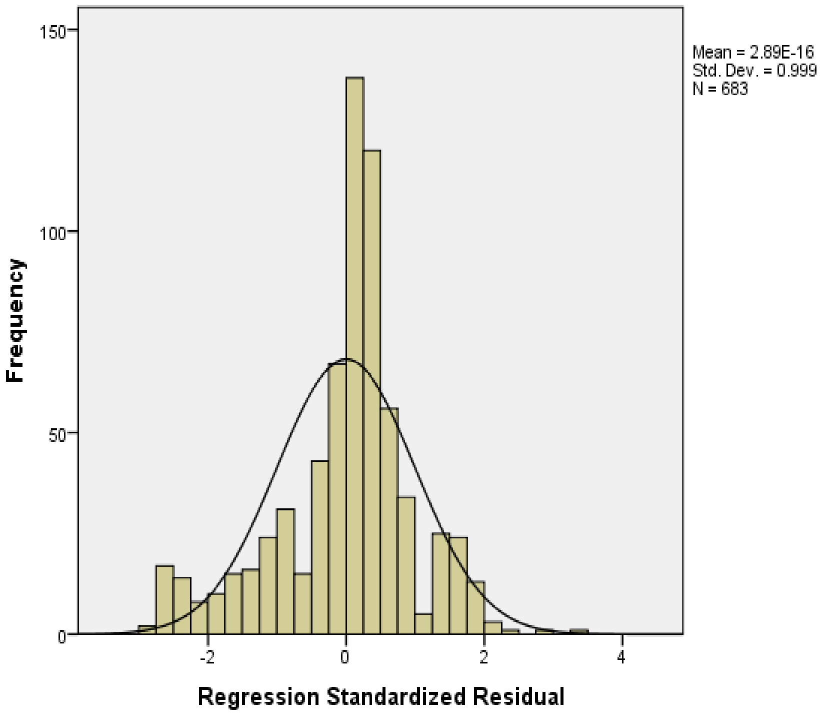

Normal P-P Plot of Regression Standardized Residual

Dependent Variable: AAD

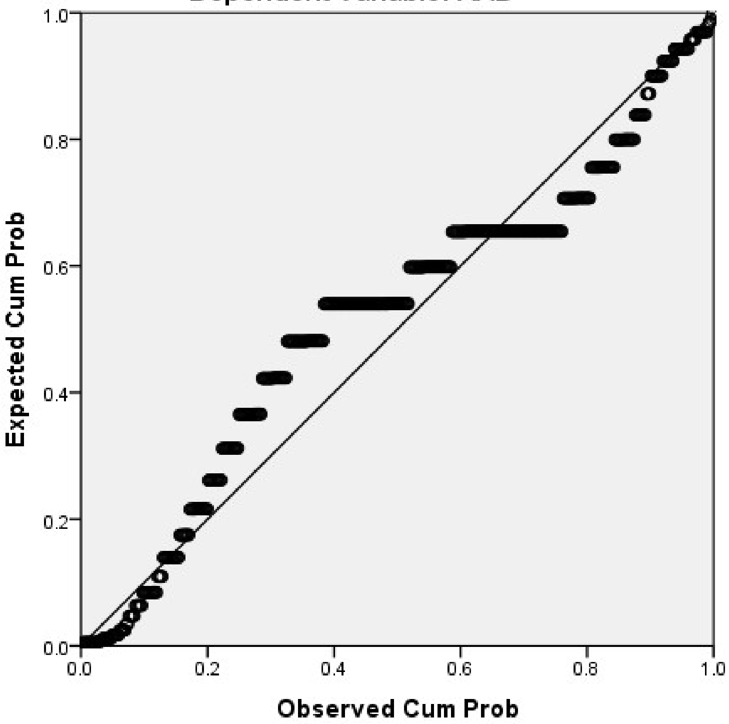

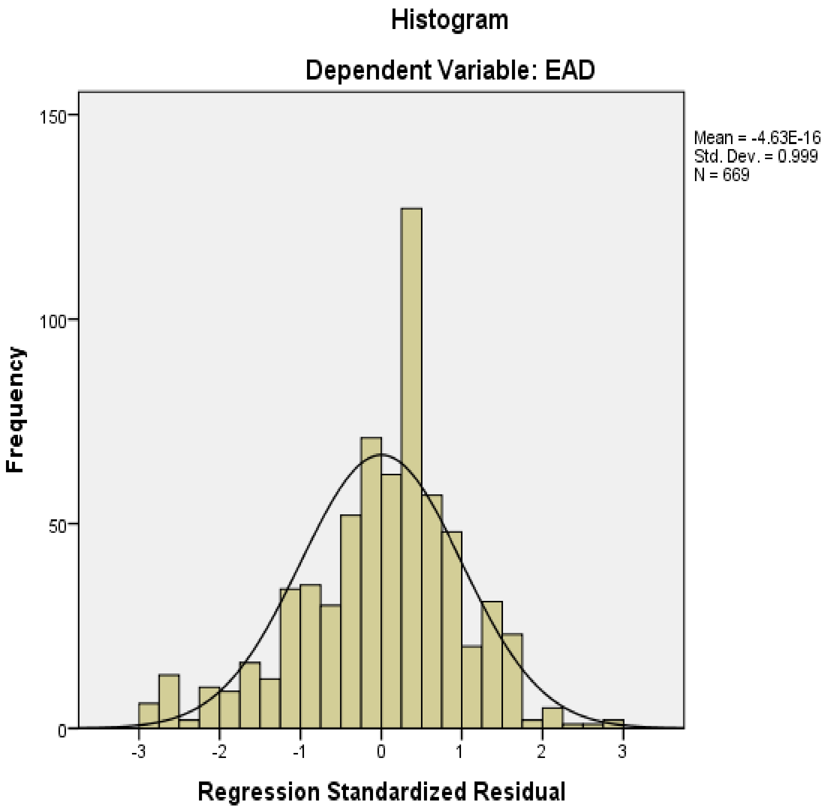
Histogram
Dependent Variable: EAD
Frequency
150
100
50
0
-3
-2
-1
0
1
2
3
Regression Standardized Residual
Mean = -4.63E-16
Std. Dev. = 0.999
N = 669

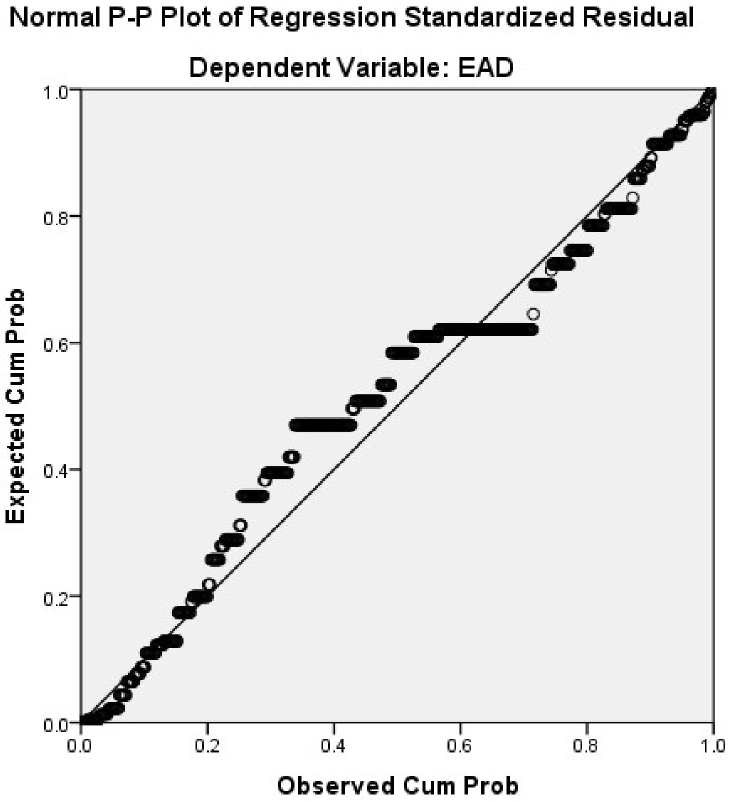
Normal P-P Plot of Regression Standardized Residual
Dependent Variable: EAD
Expected Cum Prob
1.0
0.8
0.6
0.4
0.2
0.0
0.0
0.2
0.4
0.6
0.8
1.0
Observed Cum Prob
